# Y‐Chromosome Genetic Characterization Supports the Establishment of Calving Centered Protected Areas for Tibetan Antelope Conservation

**DOI:** 10.1002/ece3.71897

**Published:** 2025-08-03

**Authors:** Shuwen Wang, Jingqing Ma, Ruotong Cheng, Jingyi Li, Xun Zhang, Zhongyuan Lin, Qing Wei, Jiarui Chen

**Affiliations:** ^1^ College of eco‐Environmental Engineering Qinghai University Xining Qinghai China; ^2^ State Key Laboratory of Plateau Ecology and Agriculture Qinghai University Xining Qinghai China

**Keywords:** population convergence, protect, Tibetan antelope, Y‐SNP, Y‐SSR

## Abstract

Tibetan antelope (*Pantholops hodgsonii*), the flagship species of the Qinghai–Tibet Plateau, is renowned for its hardiness and resistance to low oxygen. Most of the previous studies focused on mitochondria and autosomes, with fewer studies related to the Y‐chromosome. Therefore, in this study, we analyzed the Y‐chromosome genetic diversity, population structure, and historical dynamics of Tibetan antelope populations using 26 Y‐SNP loci and 5 Y‐SSR polymorphic loci. Our results revealed a nucleotide diversity of 0.00092 ± 0.00002 and a haplotype diversity of 0.843 ± 0.029 based on 26 Y‐SNPs from 14 sequences, with a total DNA sequence length of 10,675 bp. Genotyping of 123 Tibetan antelope male samples with 5 Y‐SSR loci indicated a mean observed number of alleles of 6.600, an effective number of alleles of 4.071, Shannon's Information index of 1.215, Nei's gene diversity of 0.556, and a PIC (Polymorphism Information Content) of 0.522. The population structure analysis classified all samples into three genetic populations, showing significant genetic differentiation that dates back approximately 170,000 years. However, no corresponding relationship was found between genetic populations and their geographical distribution, suggesting population convergence among Tibetan antelope populations. We inferred that population convergence facilitated genetic mixing, so that the population was able to maintain a relatively high genetic diversity after experiencing a severe hunting crisis. Given these findings, we highlight that the current model of protected areas, which are divided into administrative areas, while offering some protection, may not be optimal for the long‐term conservation of Tibetan antelope populations. Therefore, we propose to establish a system of protected areas centered around protecting calving regions, ensuring that key breeding habitats are effectively safeguarded, while simultaneously fostering natural connections and gene flow among populations, thereby providing a safer, more suitable, and coherent living environment for the Tibetan antelope.

## Graphical abstract

This study analyzed the genetic diversity, population structure, and historical dynamics of the Y chromosome of the Tibetan antelope (*Pantholops hodgsonii*), and found that its nucleotide diversity and haplotype diversity were high, and that the population was divided into three genetic populations, but there was no correspondence between the genetic groups and the geographic distribution, and the phenomenon of convergence of the populations was presumed to exist. For the conservation of the Tibetan antelope, this study suggests the establishment of a protected area system centered on the protection of lambing areas, which will promote natural linkages and gene flow among populations, and provide a safer, more suitable, and more coherent living environment for the Tibetan antelope.

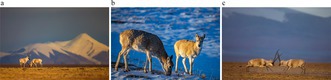

## 1 Introduction

**FIGURE 1:**
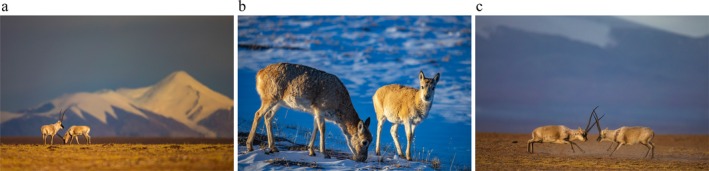
Tibetan antelope (the photos were taken and provided by Hui Qin). (a) Male Tibetan antelope; (b) Female Tibetan antelope; (c) Male Tibetan antelope.

The Qinghai–Tibet plateau (QTP), called “the Third Pole” of the Earth, is the highest plateau in the world. Due to its unique landforms and climate‐hydrological conditions, the QTP was identified as the hotspot of biodiversity and global environmental change because of its habitat and biological richness (Regmi et al. 2023). Tibetan antelope (*Pantholops hodgsonii*), the flagship species inhabiting in the QTP for centuries, has evolved adaptive mechanisms that enable them to cope with the extreme conditions of low temperature and hypoxia (Tong et al. 2013; Ma et al. 2014; Signore and Storz 2020). In the previous decades, the Tibetan antelope population suffered dramatic populations decline and population bottlenecks because of the large‐scale poaching activities for their valuable underfur. Over the last 30 years, the population of Tibetan antelopes recovered rapidly with the joint protection of the Chinese government and the international community. Despite the increase in the number of Tibetan antelopes and the downgrade of their status from “endangered” to “near‐threatened” (Zhou et al. 2007; Cao et al. 2020), they still face the risk of worsening environment caused by human activities and global warming. Figure 1a shows two male Tibetan antelopes, Figure 1b shows two female Tibetan antelopes, and Figure 1c shows male Tibetan antelopes competing for mating rights and exhibiting aggressive behavior.

Genetic diversity reflects the potential of species to cope with environmental change and evolution, and it is an important aspect of species conservation (Ellegren and Galtier 2016). Research on the Florida panther and desert tortoise has demonstrated a positive correlation between genetic diversity and fitness (Johnson et al. 2010; Scott et al. 2020). Studies based on the mtDNA showed that the Tibetan antelope population has high variation in the mitochondrial D‐loop sequence with the nucleotide diversity ranging from 0.02178 ± 0.00172 to 0.025 ± 0.10 (Ruan et al. 2005; Zhang et al. 2013). Analyses of historical population dynamics indicate that the Tibetan antelope has undergone a sustained demographic increase, accompanied by substantial population expansion throughout its evolutionary history. Genetic diversity based on autosomal SSR markers showed that the Tibetan antelope population maintained a high degree of genetic diversity, with mean allele counts ranging from 9.4 to 13.286, mean observed heterozygosity ranging from 0.810 to 0.844 ± 0.0349, and PIC values greater than 0.5 for all loci, implying a high degree of polymorphism at microsatellite loci. Genetic diversity showed an upward trend between 2003 and 2013. Paired *t*‐test showed significantly higher mean He (0.840 vs. 0.818, *p* < 0.05) and PIC (0.813 vs. 0.789, *p* < 0.05) values in Pop2013 compared to Pop2003. This may be due to migration corridors brought about by summer calving and the establishment of nature reserves, which facilitated gene flow between populations (Zhou et al. 2007; Du et al. 2016). There have been a number of studies describing the historical population dynamics of the Tibetan antelope. Analyses using distinct genetic markers have revealed divergent impacts of ice ages on the historical population dynamics of the Tibetan antelope. Although Ruan et al. (2005) proposed population expansion during the postglacial, Du et al. (2010) reported no detectable influence of the last glacial maximum (LGM) on population dynamics. It is worth noting that there is still a lack of genetic studies based on the Y chromosome in the Tibetan antelope. As a species with a high proportion of females and a polygynous mating system (Xia and Yang 2015), the genetic structure of the Tibetan antelope population and the formulation of conservation strategies need to take into account sex‐specific factors. Under this mating system, males have to compete for limited mating opportunities and face greater survival and reproduction pressure than females throughout their life cycle. The Y chromosome, as the key carrier of male sex determination and genetic information transmission, exists only in males, making their genetic diversity more vulnerable to the imbalance of male–female ratios in the population and overbreeding of dominant males, which in turn leads to a higher risk of genetic loss. Therefore, the study of Y‐chromosome genetic diversity is urgently needed to comprehensively assess the genetic status of the Tibetan antelope and to formulate scientific conservation strategies. Additionally, genetic rescue plans can be formulated in response to the potential risk of male inbreeding, thus ensuring the long‐term maintenance of the genetic diversity and health of the Tibetan antelope population.

Therefore, this study aims to comprehensively grasp the genetic diversity, population genetic structure, and population history of the male‐specific region of the Y chromosome of the Tibetan antelope through the Y‐SNP and Y‐SSR polymorphic loci, which will help us to understand more about the evolutionary history of the species and provide scientific recommendations for species conservation.

## 2 Materials and Methods

### 2.1 Sample Collection

**FIGURE 2:**
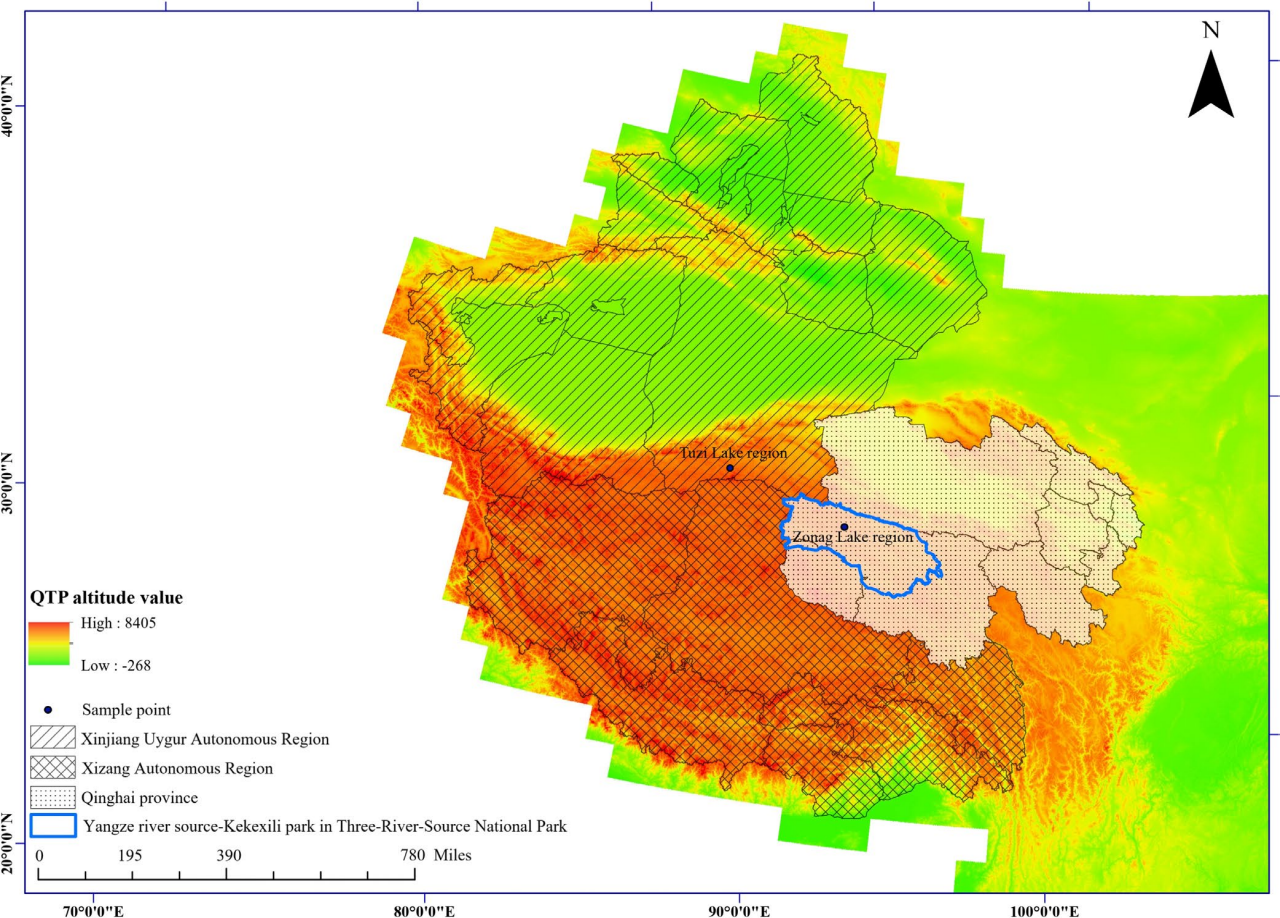
Sketch map of the sampling locations of Tibetan antelopes.

The Tibetan antelope population in China is primarily distributed across three main breeding areas: Zonag Lake lambing region in Kekexili region of Three‐River‐Source National Park in Qinghai Province, Tuzi Lake lambing region in Arjinshan Nature Reserve in Xinjiang Uygur autonomous region, and the North Changtang area spanning Xizang Autonomous Region and Xinjiang Uygur autonomous region. Fresh muscle, placenta, and skin tissues were collected in 2021 and 2022 from Zonag Lake (*n* = 214) and Tuzi Lake (*n* = 13), with non‐repetitive sampling conducted in different directions each day. Since we collected the samples in the Tuzi Lake and Zonag Lake areas, which are calving regions, the samples collected from the same calving regions were considered as a geographic population. One hundred and twenty‐three of these male Tibetan antelope samples were identified using molecular markers from the male‐specific region of the bovine Y chromosome. A sketch map of the sampling locations of Tibetan antelopes is shown in Figure 2. All samples and procedures were in accordance with the guidelines of the regulations of experiments on animals and were approved by the China Zoological Society.

### 2.2 DNA Extraction, Polymerase Chain Reaction Amplification and Sequencing

Tibetan antelopes were genotyped with 26 Y‐SNP sites and 5 Y‐SSR sites, as described in previous studies (Cheng et al. 2022; Wang et al. 2024) (Tables S1, S2). DNA extraction was performed using the chloroform‐isoamyl alcohol (24:1) method as described by Strauss et al. (Strauss 1999). The PCR reaction system consisted of 100 ng of DNA template, 1 μL each of upstream and downstream primers, 4 μL of dNTP, 5 μL of 10 × Ex Taq buffer, 0.25 μL of Ex Taq enzyme, and ddH₂O to make up a total volume of 25 μL. PCR amplification was performed with an initial denaturation at 94°C for 5 min, followed by 35 cycles of denaturation at 94°C for 30 s, annealing at X°C (X being the optimal annealing temperature for the primers) for 30 s, and extension at 72°C for 1 min, with a final extension at 72°C for 15 min. The results were detected by 1.2% agarose gel electrophoresis and then sent to GENEWIZ company for Sanger sequencing.

### 2.3 Genetic Diversity Analysis of Polymorphic Loci

Nucleotide diversity and haplotype information of Y‐SNP were calculated using DnaSP v6 (Rozas et al. 2017). Genetic diversity index and polymorphism information content (PIC) of Y‐SSR were calculated by GenAIEx v6.51 (Peakall and Smouse 2012) and PIC_CALC (Nagy et al. 2012), respectively.

### 2.4 Analysis of Phylogeny and Population Genetic Structure

Based on the polymorphic Y‐SNP sites using sheep ( *Ovis aries* ) and yak ( *Bos grunniens* ) as outgroups, the haplotype data were used to construct a phylogenetic tree of male Tibetan antelope. The phylogenetic tree based on maximum likelihood (ML) analysis was constructed by using PhyloSuite v1.2.3 (Xiang et al. 2023). The ModelFinder module and the Akaike information criterion (AIC) were used to select a best‐fit DNA substitution model, which showed that the optimal substitution model for the Y chromosome was GTR + F + G4. Finally, it was visualized by FigTree v1.4.3 (http://tree.bio.ed.ac.uk/software/figtree). Besides, it has been shown that NETWORK (Bandelt et al. 1999) can represent intraspecific evolutionary relationships more efficiently than classical phylogenetic methods, and a network diagram of male Tibetan antelope haplotypes was constructed by Network v10.2. Based on the polymorphic Y‐SSR loci, the genetic structure of the population was analyzed using Structure (Evanno et al. 2005). Given that in many species, the Y chromosome does not undergo recombination with the X chromosome, and gene flow between individuals or populations may be restricted, Admixture and No‐Admixture models were tested during the analysis. Structure v2.3 analyses were performed using the Allele Frequencies Correlated model with 3 runs per *K* value (2–8), MCMC chain length set to 50 000, with a burn‐in of 10 000 runs, and haploid mode (ploidy = 1).

According to the obtained genetic population structure, the corresponding haplotype data were exported in .arp format using DnaSP v6 and GenAIEx. Pairwise *F*_ST_ among the genetic populations was assessed for DNA sequences using Arlequin v3.0.1 (Excoffier et al. 2007). Additionally, the population genetic structure was determined via principal component analysis (PCA) using Origin 2021.

### 2.5 Estimation of Divergence Times and Demographic History

Bayesian inference (BI) was performed using BEAST v1.10.4 (Drummond et al. 2012). Sheep ( *Ovis aries* ) and cattle ( *Bos taurus* ) (Bibi 2013) were used as outgroups to estimate the time of the most recent common ancestor (tMRCA) of all haplotypes. We set up the “Strict clock” and the Yule process, and Markov Chain Monte Carlo (MCMC) analyses were run for 1 × 10^7^ generations. The log files produced by the operations were checked for the effective number of samples (ESS) for each parameter using Tracer v1.7.2 to assess the convergence of the parameters. The “TreeAnnotator” program in the BEAST package was used to annotate the tree, and 10% of trees were discarded as “burn‐in.” The dynamics of fluctuations in effective population size (Ne) of the Tibetan antelope population over historical time were modeled using the Bayesian Skyline Plot (BSP) module. The nucleotide mutation rate per nucleotide site per generation was set to 7.83 × 10^−9^ (Wang and Obbard 2023). DnaSP v6 was used to calculate mismatch distributions.

## 3 Results

### 3.1 Genetic Diversity

In this study, the amplified fragment lengths of the 26 Y‐SNPs from 14 sequences were 10,675 bp; the polymorphic sites included 21 transitions and 5 transversions. The nucleotide diversity was 0.00092 ± 0.00002, and the haplotype diversity was 0.843 ± 0.029 based on the joint analysis of 71 samples (Table 1). A total of 17 haplotypes were identified, of which seven haplotypes were rare, and the frequency of these rare haplotypes was 1.4% (Table S3). The genetic polymorphism of five Y‐SSR loci was analyzed using 123 Tibetan antelope male samples. The results showed that the mean number of alleles, effective number of alleles, mean Shannon's Information index, Nei's gene diversity, and polymorphism information content (PIC) were 6.4, 4.071, 1.215, 0.556, and 0.522, respectively (Table 2). Furthermore, 123 male Tibetan antelopes were identified into 59 haplotypes (H1–H59), of which 32 haplotypes were rare haplotypes, with a frequency of 0.8% for these rare haplotypes (Table S4).

**TABLE 1:** Genetic diversity at Y‐SNP loci of Tibetan antelope.

Gene	Number of effective sequencing samples	Number of haplotypes	Nucleotide diversity±Standard deviation	Haplotype diversity±Standard deviation
*SRYOY1*	102	2	0.00092 ± 0.00001	0.504 ± 0.008
*SNP4*	74	3	0.00060 ± 0.00005	0.479 ± 0.035
*SNP33*	84	3	0.00128 ± 0.00002	0.550 ± 0.022
*SNP43*	87	3	0.00205 ± 0.00004	0.557 ± 0.023
*SNP44*	75	3	0.00145 ± 0.00006	0.556 ± 0.024
*SNP48*	76	3	0.00078 ± 0.00002	0.617 ± 0.027
*SNP51*	81	2	0.00054 ± 0.00001	0.506 ± 0.009
*SNP54*	97	2	0.00026 ± 0.00007	0.170 ± 0.048
*SNP55*	79	3	0.00125 ± 0.00008	0.527 ± 0.021
*SNP65*	80	3	0.00056 ± 0.00004	0.563 ± 0.024
*SNP71*	73	2	0.00097 ± 0.00004	0.495 ± 0.020
*SNP73*	82	4	0.00127 ± 0.00005	0.595 ± 0.028
*SNP74*	90	2	0.00016 ± 0.00006	0.106 ± 0.043
*SNP75*	92	3	0.00141 ± 0.00007	0.556 ± 0.030
Total	71	17	0.00092 ± 0.00002	0.843 ± 0.029

**TABLE 2:** Genetic diversity at Y‐SSR loci of Tibetan antelope.

Loci	Na	Ne	I	H	PIC
*SSR2*	5	3.057	1.206	0.673	0.607
*SSR18*	7	4.209	1.586	0.762	0.731
*SSR20*	2	1.216	0.321	0.177	0.161
*SSR37*	2	1.437	0.482	0.304	0.258
*SSR44*	17	9.986	2.529	0.900	0.892
Mean	6.600	3.981	1.225	0.563	0.530

Abbreviations: h, Nei's gene diversity; I, Shannon's information index; Na, observed number of alleles; Ne, effective number of alleles; PIC, polymorphism information content.

### 3.2 Population Genetic Structure

**FIGURE 3:**
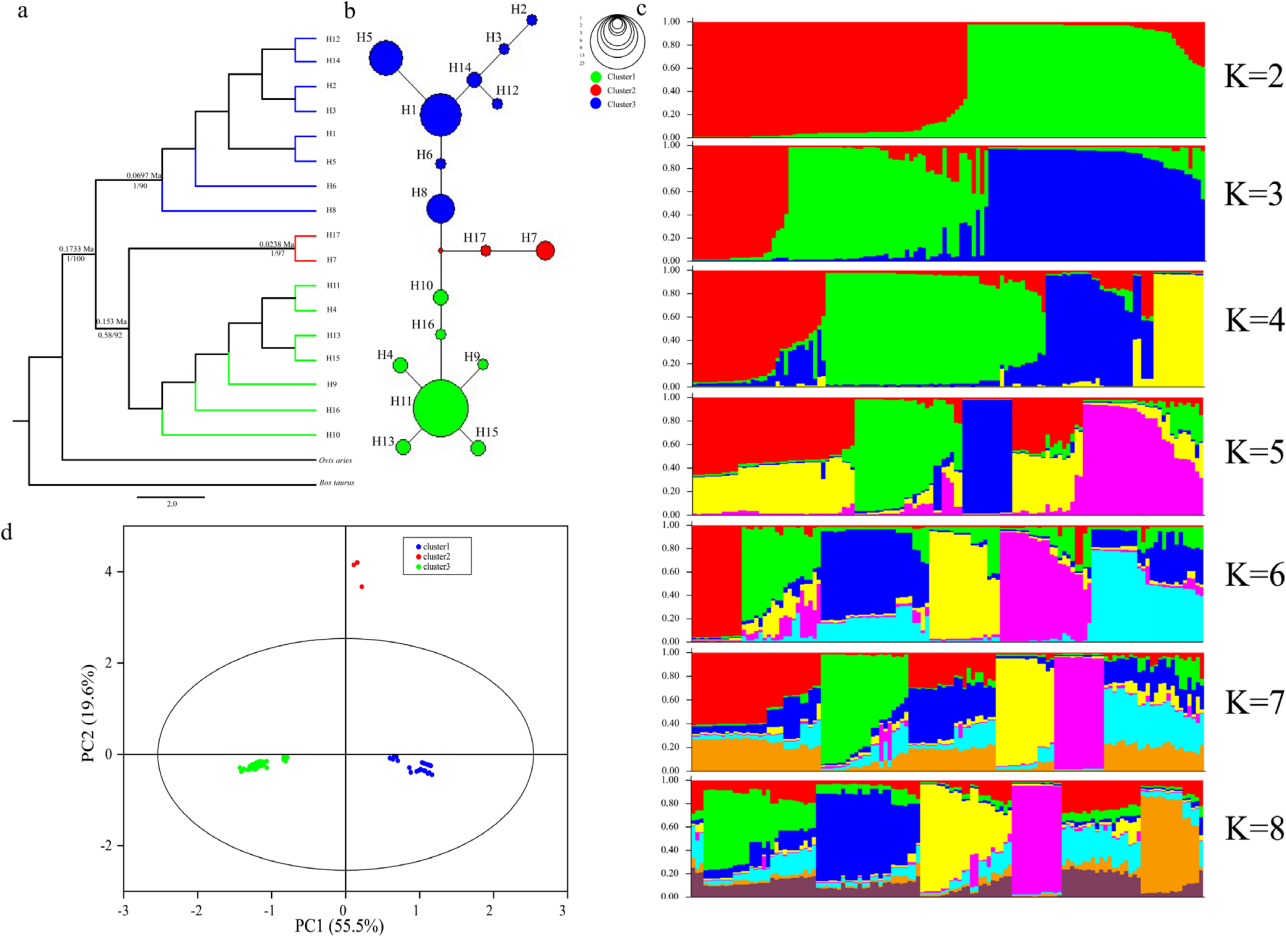
Population genetic structure in Tibetan antelope. (a) Phylogenetic tree. The numbers on the branch line are the divergence times estimated using BEAST, the numbers on the left below the branch line correspond to the support rates from the BI analysis, and the numbers on the right indicate ML bootstrap support rates based on 1000 repetitions. (b) Network of the 17 haplotypes. Circle sizes correspond to the number of samples. (c) Results of Bayesian individual‐based clustering. (d) PCA plot based on Y‐SNP and Y‐SSR among three genetic populations.

The phylogenetic tree (Figure 3a) and network diagram (Figure 3b) were constructed based on the haplotype data. All haplotypes diverged into three clades in the ML tree, and the network diagram was greatly consistent with the ML tree. Based on the structure of the network diagram, H11 was hypothesized to be an ancient haplotype with the highest haplotype frequency of 32.4%.

Population genetic structure was jointly analyzed for five polymorphic Y‐SSR loci in 123 Tibetan antelope samples. As Structure is a calculation method based on the Bayesian model, during the analysis, a likelihood value is obtained for each *K*‐value simulation, and the *K*‐value with the maximum likelihood value is of particular interest. In both Admixture and No‐Admixture models, when *K* = 3, the likelihood reached its maximum, indicating that the simulated result corresponding to this *K* value was the closest to the true genetic structure of the population. Moreover, the results showed that the *Q* values of all individuals were greater than 0.6, suggesting a relatively homogeneous genetic background. Notably, the clustering patterns produced by the two models were nearly identical (Figure 3c and Figure S1), demonstrating the robustness of the inferred population structure. Based on these results, the 123 male Tibetan antelope samples were divided into three genetic clusters. All samples were grouped into three clusters in PCA based on Y‐SNP and Y‐SSR (Figure 3d).

### 3.3 Genetic Differentiation Among the Genetic Populations

In summary, the Tibetan antelope populations were separated into three genetic populations combined with all the population genetic structure results. To clearly understand their genetic differentiation relationships among the three genetic populations of Tibetan antelope, we analyzed the genetic differentiation index. In this section, each genetic cluster was classified as a population. For example, the blue cluster was denoted as Pop 1, the red cluster was denoted as Pop 2, and the green cluster was denoted as Pop 3. The genetic differentiation results among the three genetic populations are presented in Table 3, which indicated a significant genetic differentiation.

**TABLE 3:** Pairwise comparisons of genetic differentiation (*F*_ST_) based on haplotype frequencies of Y‐SNP and Y‐SSR loci.

	Populations	Pop1	Pop2	Pop3
Y‐SNP	Pop1	—		
	Pop2	0.31021 (*p* < 0.01)	—	
	Pop3	0.35846 (*p* < 0.01)	0.48992 (*p* < 0.01)	—
Y‐SSR	Pop1	—		
	Pop2	0.08082 (*p* < 0.05)	—	
	Pop3	0.04916 (*p* < 0.01)	0.10205 (*p* < 0.05)	—

### 3.4 Inferences of Divergence Times and Demographic History

Estimation of divergence times and analysis of historical population dynamics, as revealed in the tMRCA analysis in BEAST (Figure 3a), indicates that genetic differentiation of the Tibetan antelope population occurred over the past 0.1733 Ma BP (95% HPD: 0.1141–0.2345 Ma). Additionally, Y‐1 and Y‐2 diverged approximately 0.153 Ma BP (95% HPD: 0.0899–0.2218 Ma). This suggests that the ancestral Tibetan antelope underwent genetic differentiation on the paternal lineage at an early stage in its evolutionary history.

**FIGURE 4:**
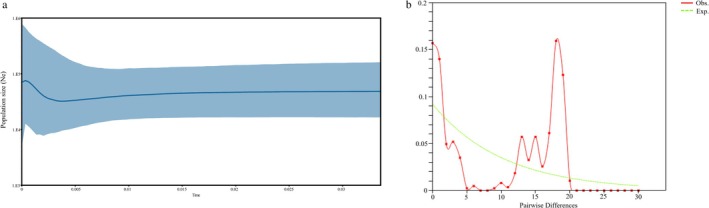
Results of coalescent analysis using BEAST and mismatch distribution analysis. (a) Bayesian skyline plot derived from all samples of the chirus. The *x*‐axis is in units of million years in the past and the *y*‐axis is Ne × μ (effective population size × mutation rate per generation). Smoothed curves show mean and 95% of the highest posterior density (blue‐shaded region) values for effective population size over time. (b) Mismatch distribution analyses of all samples.

Bayesian skyline analyses yielded historical dynamics of the Tibetan antelope population, as shown in Figure 4a. The male Tibetan antelope population tended to remain relatively stable until 10 kya BP, with a slow contraction of the population between 10 kya and 3.5 kya, and a marked expansion of the population between 3.5 kya and 0.5 kya, followed by population contraction occurring in the past 500 years. The results of the mismatch distribution plot showed multiple peaks, which may have been indicative of populations experiencing more complex dynamics (Figure 4b).

## 4 Discussion

Genetic diversity in the male‐specific region of the Tibetan antelope Y chromosome was lower than that of both the mitochondrial control region and autosomes in the present study (Table S5), which is consistent with the results of many studies (Malaspina et al. 1990; Jaruzelska et al. 1999; Sachidanandam et al. 2001). Despite this, a comparison of the genetic diversity of the Tibetan antelope with that of other species suggests that it still has relatively high genetic diversity (Table S6). Meanwhile, when comparing the genetic diversity of the Tibetan antelope with that of wildlife such as Takin ( *Budorcas taxicolor* ), Hainan gibbon ( *Nomascus hainanus* ), South China tiger (*Panthera tigris ssp. amoyensis*), and Yangtze crocodile ( *Alligator sinensis* ), it is found that even after the worst crisis caused by poaching, the genetic diversity of the Tibetan antelope is still relatively high compared to these species (Guo et al. 2021; Li et al. 2023; Wang et al. 2023; Yang et al. 2023). To a certain extent, this is a reflection of the remarkable conservation effect of the Tibetan antelope in recent decades.

All Tibetan antelope male samples were divided into three genetic groups, and analyses showed significant genetic differentiation between the three genetic populations. However, there was no corresponding relationship between the genetic populations and their geographical distribution. Based on the divergence time, it was indicated that the earliest genetic differentiation of the population occurred approximately 0.1733 Ma ago (95% HPD: 0.1141–0.2345 Ma), which is basically consistent with the occurrence of the Gonghe Movement (approximately 150, 000 years ago) (Li and Fang 1999). The differentiation times of Tibetan antelope populations based on the male‐specific region of the Y chromosome are basically the same as those obtained by previous authors based on mitochondrial sequences (Chen et al. 2019). During the Gonghe Movement, the QTP underwent dramatic and heterogeneous tectonic uplift (Shi et al. 1999), reaching its present‐day height and forming new geographic barriers (Chang et al. 2016). The environmental record since then provides complete coverage of the Last Glacial Cycle (Shi et al. 1999). These newly formed geographic barriers, along with the climatic changes during this period, have created the possibility for population genetic differentiation among species on the QTP (Qi et al. 2006; Favre et al. 2015). While we initially speculated that the uplift of the Tibetan Plateau caused by the Gonghe Movement had been the direct cause of the divergence of male Tibetan antelope populations, it is important to consider alternative hypotheses to strengthen this argument. Climate oscillations, for instance, could also have played a significant role. The environmental records during the Gonghe Movement period and beyond revealed a series of complex climate changes. The Guliya ice core records showed that there were three warm peaks at 80 ka, 95 ka, and 120 ka, with temperatures 3°C, 0.9°C, and 5°C higher than the modern average temperature, respectively (Shi et al. 1999). Following these warm peaks, the last glacial maximum suddenly arrived, and the temperature dropped by about 12°C within 3 ka. The lowest temperature during the last glacial maximum occurred around 23 ka BP, with an average temperature approximately 9°C lower than the modern average temperature (Chen 2018). Tianshuihai Lake core records showed that the QTP was the warmest and wettest at 120 ka BP, when the lake was closed. The QTP experienced warmer and wetter conditions, which may have expanded grassland habitats and facilitated greater mobility across the plateau, and such conditions may have contributed to the formation of temporary migratory corridors that allowed gene flow between otherwise isolated regions. At the beginning of the cold—warm transition between the 110 ka BP cold period and the 95 ka BP warm period, a high lake level appeared on the QTP. During the 55–30 ka BP stage, there were multiple fluctuations in climate and lake levels, with a warm and humid event lasting about 3 ka (Chen 2018). After 30 ka BP, the climate became cold and wet, forming a sub–high lake evel (Zheng et al. 2005). These climate oscillations could have led to changes in the distribution of resources, such as food and water, on the QTP. For Tibetan antelopes, such changes might have forced them to migrate to more suitable habitats. The resulting geographical isolation between these new habitats could have then promoted genetic differentiation among different populations of Tibetan antelopes. The divergence of clades Y‐1 and Y‐2 occurred approximately 0.153 Ma ago (95% HPD: 0.0899–0.2218 Ma), again coinciding with the onset of the Gonghe Movement. It is hypothesized that the rapid uplift of the Tibetan Plateau, in conjunction with the climatic changes during this period, may have played an important role in the differentiation and integration of Tibetan antelope populations. The combined effects of tectonic uplift and climate oscillations could explain the lack of correspondence between the genetic structure and the geographical distribution patterns of the Tibetan antelope populations that were analyzed (Table S3).

The results of the Bayesian Skyline Plot (BSP) indicate that the first population contraction of male Tibetan antelopes occurred during the Holocene Great Warm Period on the Tibetan Plateau, spanning approximately from 10, 000 to 3500 years ago. During this period, the dominant vegetation on the Tibetan Plateau transitioned to alpine forest scrub (Tang and Li 2001). However, the Tibetan antelope prefers the alpine desert, and the alpine forest scrub environment is not suitable for their survival (Ginsberg et al. 2000; Zhuge et al. 2014). In order to survive, Tibetan antelopes had to migrate northward, which to some extent promoted population integration but may have also contributed to the decline in their population size. Between 500 and 3500 years ago, the Tibetan antelope underwent its first population expansion. During this period, the climate of the QTP was relatively stable, with vegetation distribution almost identical to that of today. Consequently, the Tibetan antelope's population distribution area expanded, and its population size increased continuously. Since 500 years ago, a second population contraction has occurred in the Tibetan antelope, which may be attributed to human activities leading to a decline in population numbers. Single peaks in the mismatch distribution plot indicated that the population had experienced expansion, while multiple peaks may have suggested more complex population dynamics, including multiple expansions, contractions, or bottleneck events. The results of the mismatch distribution plot showed a multi‐peak status, which was consistent with the historical population dynamics of the Tibetan antelope (Rogers and Harpending 1992).

At the beginning of the 20th century, there were about 1 million Tibetan antelopes on the Tibetan Plateau, but by 2003 their numbers had dropped to about 50 000 (Wang et al. 2022). Although the Tibetan antelope had lost 95% of its original population, it still maintained a high level of genetic diversity, and its population was recovering rapidly. We suggested that this was partly due to two factors. One was population convergence resulting from vegetation succession associated with past geological and climatic events, as well as gene flow stemming from the unique migratory behavior of the Tibetan antelope. The other was the conservation efforts for the Tibetan antelope in the last 20 years (Ruan et al. 2005; Kuang 2021). However, this did not mean that our level of protection for the Tibetan antelope could be reduced. The Tibetan antelope population harbored many rare haplotypes, which were crucial yet highly susceptible to loss if the animals that carried them died. Moreover, the effects of human activities and global warming on the Tibetan antelope could not be overlooked.

In conclusion, the male‐specific region of the Y chromosome of the Tibetan antelope retains relatively high genetic diversity, indicating that conservation efforts for the Tibetan antelope have been effective in recent decades. However, the high frequency of rare haplotypes and human activities are factors that necessitate continued conservation efforts for the Tibetan antelope. Additionally, the population convergence driven by geologic and climatic events and calving migration was suggested to play essential roles in the genetic diversity maintenance. However, the current protected areas are all divided by the boundaries of administrative districts. Although this division is convenient for management, the distribution of species often transcends these boundaries, crossing multiple administrative districts to form continuous habitats or migratory paths. Consequently, the pure reliance on administrative district boundaries for dividing protected areas often leads to discontinuity, artificially severing key ecological areas or species migration corridors, which is not conducive to species conservation in the long run. Therefore, against the backdrop of global climate change and the escalating loss of biodiversity, we propose to construct a conservation system centered on calving regions. Although the construction of protected areas may face challenges in terms of coordination between different sectors, this system will rather be based on the natural distribution and ecological needs of species to delineate the scope of protection. Specifically, we should conduct comprehensive surveys and assessments of the species' calving regions, identify their critical ecological zones and migration paths, and delineate the boundaries of protected areas accordingly. Meanwhile, we need to strengthen inter‐administrative cooperation and coordination to ensure the continuity and integrity of these protected areas. It is equally important to avoid fragmentation and duplication of conservation efforts caused by artificial administrative divisions.

## Author Contributions

**Shuwen Wang:** data curation (equal), investigation (equal), writing – original draft (equal), writing – review and editing (equal). **Jingqing Ma:** data curation (equal), methodology (equal), writing – review and editing (equal). **Ruotong Cheng:** data curation (equal), methodology (equal), writing – review and editing (equal). **Jingyi Li:** data curation (equal), methodology (equal), writing – review and editing (equal). **Xun Zhang:** data curation (equal), writing – review and editing (equal). **Zhongyuan Lin:** data curation (equal), writing – review and editing (equal). **Qing Wei:** conceptualization (equal), investigation (equal), resources (equal), writing – review and editing (equal). **Jiarui Chen:** conceptualization (equal), funding acquisition (equal), investigation (equal), resources (equal), writing – review and editing (equal).

## Conflicts of Interest

The authors declare no conflicts of interest.

## Supporting information


**Data S1:** ece371897‐sup‐0001‐Supinfo.docx.

## Data Availability

The data supporting the findings of this study are available in the Dryad Digital Repository under the permanent DOI: https://doi.org/10.5061/dryad.4b8gthtrs. The dataset is undergoing curation and is not yet publicly accessible. For the purpose of peer review, a temporary reviewer sharing link has been provided to facilitate access to the dataset.
