# Supplementary material for: Y‐Chromosome Genetic Characterization Supports the Establishment of Calving Centered Protected Areas for Tibetan Antelope Conservation
Source: Ecol Evol. 2025 Aug 3;15(8):e71897. doi: 10.1002/ece3.71897 (PMC12318635; doi:10.1002/ece3.71897)
Supplement: Supplementary file 1 — Data S1: ece371897‐sup‐0001‐Supinfo.docx. [file ECE3-15-e71897-s001.docx]

Table S1. Primer pairs of 26 Y-SNP sites in Tibetan antelope

| Primer | Sequence | Ta℃ | References |
| --- | --- | --- | --- |
| *SRYOY1* | F: TCAGTAGCTTAGGTACATTCA | 56 | Meadows et al., 2004 |
|  | R: GTGCTACATAAATATGATCTGC |  |  |
| *SNP4* | F：CTTCTGCAGCACTCAATGGA | 62 | Wang et al., 2024 |
|  | R：CCTTGGCTTGACGGACCATA |  |  |
| *SNP33* | F：AACTGCTTATCTGGGTGCCT | 60 | Wang et al., 2024 |
|  | R：TCCTTGGCCACTGGAAAAGA |  |  |
| *SNP43* | F：TCTCCAACCGCTGAACTGAT | 60 | Wang et al., 2024 |
|  | R：AGCCTGAATTGAGATGCCCT |  |  |
| *SNP44* | F：GCCCTTCAAACCAATCTGCA | 60 | Wang et al., 2024 |
|  | R：TCATCCTTTGGCCTTCCCTT |  |  |
| *SNP48* | F：GGTCGTCTGTATCTGGGATTCT | 63.3 | Wang et al., 2024 |
|  | R：CTCAGTCCACACATGTTGCTAC |  |  |
| *SNP51* | F：TGAACGGGTACACACAATGC | 60 | Wang et al., 2024 |
|  | R：AGGCCTGGTTGCACATTAGA |  |  |
| *SNP54* | F：TGCCAACCCCAGCTAGTTAT | 60 | Wang et al., 2024 |
|  | R：AAGCATCAATTACTCGGCGC |  |  |
| *SNP55* | F：GTTGTCACTCTTGTCTTGTTCTC | 61.4 | Wang et al., 2024 |
|  | R：TACTCTTTCTGTCCCCCTCTGA |  |  |
| *SNP65* | F：GGAAAGCCAATTAAGCCGCT | 60 | Wang et al., 2024 |
|  | R：TCCCCCATGCCACATAGTTT |  |  |
| *SNP71* | F：AGTGTCGCATCTGTCAGTGT | 65 | Wang et al., 2024 |
|  | R：ATTGTCACACAGGAGGCAGA |  |  |
| *SNP73* | F：AGACAACCCAAGAGATGCCA | 60 | Wang et al., 2024 |
|  | R：ACATAGCTGTCCGGTGTTCT |  |  |
| *SNP74* | F：GCCACCAATTAGCCTGCAAT | 60 | Wang et al., 2024 |
|  | R：TTAGAGTTGGCTGGGCTCAA |  |  |
| *SNP75* | F：ACAGCTGTTCATGGCAAGTG | 60 | Wang et al., 2024 |
|  | R：AGTTCAACCCGCAGCCTATT |  |  |

Table S2. Primer pairs of 5 Y-SSR loci in Tibetan antelope

| loci | Core sequence | Primer | Ta℃ | References |
| --- | --- | --- | --- | --- |
| *SSR2* | (TA)_n_ | F：GCTGGGTAAACCACTTTGGA | 59.9 | Wang et al., 2024 |
|  |  | R：ATGGTGATGGACAGGGAGAC |  |  |
| *SSR18* | (GT)_n_ | F：CCAAGAGTTGGCCACATAAA | 59.0 | Wang et al., 2024 |
|  |  | R：TTACATGCAGCCAAGAAAACA |  |  |
| *SSR20* | (AT)_n_ | F：TTGTGAAAATCTGGGTGAATTTT | 59.8 | Wang et al., 2024 |
|  |  | R：GAGGAAGCACCATATTTTACGTTT |  |  |
| *SSR37* | (ATAC)_n_ | F：CATGGCTTGAGTCATCATCTG | 59.8 | Wang et al., 2024 |
|  |  | R：AAACTAGGGTCATGGCCTCC |  |  |
| *SSR44* (AAAG)_n_ | | F：ATCTTGTCACAGGTGGGGTC | 59.8 | Wang et al., 2024 |
|  |  | R：CATACAGCCTCTGCTTGCTG |  |  |

Table S3. 17 haplotypes determined based on 26 Y-SNP markers of Tibetan antelope

| Haplotypes | Frequency | Sample location | No. of individuals |
| --- | --- | --- | --- |
| H1 | 0.183 | Xinjiang | 2 |
|  |  | Qinghai | 11 |
| H2 | 0.014 | Xinjiang | 1 |
| H3 | 0.014 | Xinjiang | 1 |
| H4 | 0.028 | Xinjiang | 2 |
| H5 | 0.127 | Xinjiang | 2 |
|  |  | Qinghai | 7 |
| H6 | 0.014 | Qinghai | 1 |
| H7 | 0.042 | Qinghai | 3 |
| H8 | 0.085 | Qinghai | 6 |
| H9 | 0.014 | Qinghai | 1 |
| H10 | 0.028 | Qinghai | 2 |
| H11 | 0.324 | Qinghai | 23 |
| H12 | 0.014 | Qinghai | 1 |
| H13 | 0.028 | Qinghai | 2 |
| H14 | 0.028 | Qinghai | 2 |
| H15 | 0.028 | Qinghai | 2 |
| H16 | 0.014 | Qinghai | 1 |
| H17 | 0.014 | Qinghai | 1 |

Table S4. 59 haplotypes determined based on 5 Y-SSR markers of Tibetan antelope

| haplotypes | *SSR2* | *SSR18* | *SSR20* | *SSR37* | *SSR44* | No. of individuals | Frequency |
| --- | --- | --- | --- | --- | --- | --- | --- |
| H1 | 172 | 264 | 269 | 162 | 254 | 6 | 0.049 |
| H2 | 168 | 264 | 269 | 162 | 230 | 2 | 0.016 |
| H3 | 168 | 266 | 269 | 162 | 223 | 7 | 0.057 |
| H4 | 168 | 266 | 269 | 162 | 238 | 2 | 0.016 |
| H5 | 172 | 268 | 269 | 162 | 254 | 1 | 0.008 |
| H6 | 168 | 268 | 269 | 162 | 226 | 6 | 0.049 |
| H7 | 178 | 260 | 271 | 158 | 212 | 2 | 0.016 |
| H8 | 170 | 260 | 271 | 158 | 234 | 7 | 0.057 |
| H9 | 170 | 268 | 269 | 162 | 238 | 1 | 0.008 |
| H10 | 168 | 266 | 269 | 162 | 220 | 2 | 0.016 |
| H11 | 168 | 266 | 269 | 162 | 216 | 2 | 0.016 |
| H12 | 170 | 262 | 269 | 162 | 238 | 2 | 0.016 |
| H13 | 168 | 266 | 269 | 162 | 226 | 12 | 0.098 |
| H14 | 170 | 264 | 269 | 158 | 250 | 1 | 0.008 |
| H15 | 170 | 262 | 269 | 158 | 246 | 2 | 0.016 |
| H16 | 170 | 262 | 269 | 158 | 242 | 3 | 0.024 |
| H17 | 168 | 266 | 269 | 162 | 201 | 4 | 0.033 |
| H18 | 170 | 266 | 269 | 162 | 230 | 2 | 0.016 |
| H19 | 172 | 264 | 269 | 162 | 260 | 2 | 0.016 |
| H20 | 168 | 260 | 269 | 162 | 223 | 1 | 0.008 |
| H21 | 168 | 264 | 269 | 162 | 223 | 4 | 0.033 |
| H22 | 172 | 268 | 269 | 162 | 250 | 1 | 0.008 |
| H23 | 170 | 262 | 269 | 158 | 230 | 1 | 0.008 |
| H24 | 170 | 266 | 269 | 162 | 226 | 2 | 0.016 |
| H25 | 170 | 262 | 269 | 162 | 230 | 2 | 0.016 |
| H26 | 170 | 266 | 269 | 162 | 220 | 1 | 0.008 |
| H27 | 172 | 268 | 269 | 162 | 268 | 1 | 0.008 |
| H28 | 174 | 268 | 269 | 162 | 234 | 2 | 0.016 |
| H29 | 172 | 264 | 269 | 162 | 256 | 4 | 0.033 |
| H30 | 172 | 268 | 269 | 162 | 234 | 1 | 0.008 |
| H31 | 170 | 268 | 269 | 162 | 234 | 1 | 0.008 |
| H32 | 170 | 262 | 269 | 162 | 234 | 1 | 0.008 |
| H33 | 168 | 268 | 269 | 162 | 230 | 1 | 0.008 |
| H34 | 172 | 266 | 269 | 162 | 250 | 2 | 0.016 |
| H35 | 172 | 260 | 271 | 158 | 234 | 3 | 0.024 |
| H36 | 170 | 264 | 269 | 162 | 234 | 1 | 0.008 |
| H37 | 168 | 0 | 269 | 162 | 212 | 1 | 0.008 |
| H38 | 172 | 266 | 269 | 162 | 228 | 1 | 0.008 |
| H39 | 168 | 264 | 0 | 162 | 223 | 1 | 0.008 |
| H40 | 172 | 266 | 269 | 162 | 242 | 1 | 0.008 |
| H41 | 172 | 266 | 269 | 162 | 220 | 1 | 0.008 |
| H42 | 170 | 262 | 269 | 162 | 242 | 1 | 0.008 |
| H43 | 170 | 264 | 269 | 162 | 250 | 1 | 0.008 |
| H44 | 170 | 264 | 269 | 162 | 230 | 1 | 0.008 |
| H45 | 170 | 268 | 269 | 162 | 242 | 1 | 0.008 |
| H46 | 170 | 264 | 269 | 162 | 246 | 2 | 0.016 |
| H47 | 170 | 264 | 269 | 162 | 223 | 1 | 0.008 |
| H48 | 168 | 264 | 269 | 162 | 226 | 4 | 0.033 |
| H49 | 168 | 266 | 269 | 162 | 230 | 1 | 0.008 |
| H50 | 170 | 266 | 269 | 158 | 256 | 1 | 0.008 |
| H51 | 170 | 264 | 269 | 158 | 246 | 1 | 0.008 |
| H52 | 172 | 266 | 269 | 162 | 246 | 1 | 0.008 |
| H53 | 170 | 270 | 269 | 162 | 216 | 1 | 0.008 |
| H54 | 172 | 258 | 269 | 162 | 230 | 1 | 0.008 |
| H55 | 170 | 264 | 269 | 162 | 238 | 1 | 0.008 |
| H56 | 172 | 268 | 269 | 162 | 226 | 1 | 0.008 |
| H57 | 172 | 266 | 269 | 162 | 254 | 1 | 0.008 |
| H58 | 170 | 260 | 269 | 158 | 242 | 2 | 0.016 |
| H59 | 172 | 270 | 269 | 162 | 250 | 1 | 0.008 |

Table S5. Tibetan Antelope Genetic Diversity Comparison Based on Three Markers

| Genetic makers | Nucleotide diversity | Haplotype diversity | PIC | I | Data resource references |
| --- | --- | --- | --- | --- | --- |
| mtDNA control region | 0.02178 ± 0.00172 | 0.997±0.004 | — |  | (Ruan et al., 2005) |
|  | 0.025±0.10 | 0.991±0.003 | — |  | (Zhang et al., 2013) |
| Autosomal microsatellite loci | — | — | 0.818 | 1.990 | (Zhou et al., 2007) |
|  | — | — | 0.813 | 2.114 | (Du et al., 2016) |
| Y-chromosome male specific region | 0.00092±0.00002 | 0.843±0.029 | 0.530 | 1.225 | This study |

Table S6. Summary of genetic diversity parameters of Y chromosome of several mammals

| Species | PIC | Nucleotide diversity  ±Standard Deviation | Haplotype diversity  ±Standard Deviation | Data resource references |
| --- | --- | --- | --- | --- |
| Sheep (*Ovis aries*) | — | 0.00009 | — | Meadows et al., 2004 |
| Domestic yak (*Bos grunniens*) | — | 0.00017±0.00003 | — | Li et al. 2014 |
| European rabbit (*Oryctolagus cuniculus*) | — | 0.00134 | — | Geraldes et al., 2005 |
| Surti buffalo | — | — | 0.600±0.215 | Deswal et al., 2022 |
| Murrah buffalo |  |  | 0.899±0.024 | Deswal et al., 2022 |
| white yak (*Bos grunniens*) | — | — | 0.756 7 ± 0.0233 | Luo et al., 2022 |
| Luxi cattle | 0.679 | — | — | Xin et al., 2011 |
| cross breed Simmental× Qinchuan | 0.464 | — | — | Xin et al., 2011 |
| Jinnan cattle | 0.503 | — | — | Xin et al., 2011 |
| Tibetan antelope（*Pantholops hodgsonii*） | 0.522 | 0.00092±0.00002 | 0.843±0.029 | this study |


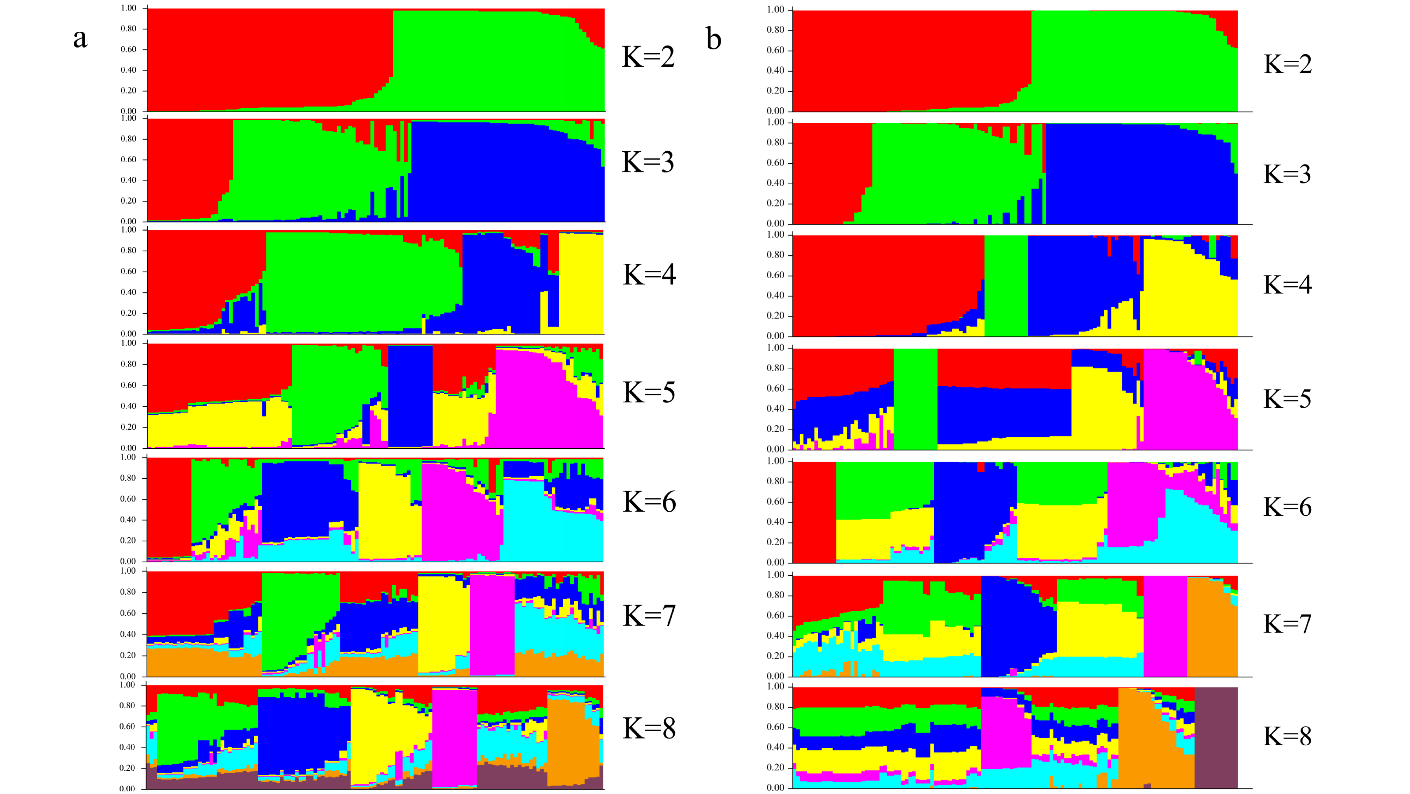


Supplementary Figure S1 STRUCTURE analysis under Admixture model and No-Admixture model

1. Analysis of admixture model b. Analysis of No-Admixture model

References:

Deswal S, Singh S, Chandrashekaraiah J, Ganguly I, Dixit SP. 2022. Identification of Y chromosome haplogroup and estimation of genetic diversity in Indian buffalo breeds using Y specific STR markers. AGRICULTURE, DAIRY & ANIMAL SCIENCE 41:183-193.

Du Y, Zou X, Xu Y, Guo X, Li S, Zhang X, Su M, Ma J, Guo S. 2016. Microsatellite Loci Analysis Reveals Post-bottleneck Recovery of Genetic Diversity in the Tibetan Antelope. Sci Rep 6:35501.

Geraldes A, Rogel-Gaillard C, Ferrand N. 2005. High levels of nucleotide diversity in the European rabbit (Oryctolagus cuniculus) SRY gene. Anim Genet 36:349-351.

Li R, Wang SQ, Xu SY, Huang JP, Wang FQ, Ma ZJ, Dang RH, Lan XY, Chen H, Lei CZ. 2014. Novel Y-chromosome polymorphisms in Chinese domestic yak. Anim Genet 45:449-452.

Luo J, Wei X, Liu W, Chen S, Ahmed Z, Sun W, Lei C, Ma Z. 2022. Paternal genetic diversity, differentiation and phylogeny of three white yak breeds/populations in China. Sci Rep 12:19331.

Meadows JR, Hawken RJ, Kijas JW. 2004. Nucleotide diversity on the ovine Y chromosome. Anim Genet 35:379-385.

Ruan X-D, He P-J, Zhang J-L, Wan Q-H, Fang S-G. 2005. Evolutionary history and current population relationships of the chiru (*Pantholops hodgsonii*) inferred from mtDNA variation. Journal of Mammalogy 86:881-886.

Wang S, Wang D, Cheng R, Chen Y, Li J, Chen Y, Chen J, Wei Q. 2024. Genome-Sequencing-Based Screening of Polymorphic Genetic Loci in the Y Chromosome of *Pantholops hodgsonii*. Sichuan Journal of Zoology 43:24-33.

Xin YP, Zan LS, Wang YH, Liu YF, Tian WQ, Fan YY. 2011. Polymorphism of bovine Y-STR UMN0929 and its correlation with carcass traits in five Chinese beef cattle populations. Mol Biol Rep 38:411-416.

Zhang F, Jiang Z, Xu A, Zeng Y, Li C. 2013. Recent geological events and intrinsic behavior influence the population genetic structure of the chiru and tibetan gazelle on the tibetan plateau. PLoS One 8:e60712.

Zhou H, Li D, Zhang Y, Yang T, Liu Y. 2007. Genetic diversity of microsatellite DNA loci of Tibetan antelope (Chiru, *Pantholops hodgsonii*) in Hoh Xil National Nature Reserve, Qinghai, China. J Genet Genomics 34:600-607.
